# The stress polarity pathway: AMPK ‘GIV’-es protection against metabolic insults

**DOI:** 10.18632/aging.101179

**Published:** 2017-02-15

**Authors:** Pradipta Ghosh

**Affiliations:** ^1^ Departments of Medicine and Cellular and Molecular Medicine, University of California at San Diego, La Jolla, CA 92093, USA

**Keywords:** LKB1, AMP-kinase, epithelial tight junctions, gut barrier, metabolic syndrome, heterotrimeric G proteins, energetic stress

## Abstract

Loss of cell polarity impairs organ development and function; it can also serve as one of the first triggers for oncogenesis. In 2006-2007 two groups simultaneously reported the existence of a special pathway for maintaining epithelial polarity in the face of environmental stressors. In this pathway, AMPK, a key sensor of metabolic stress stabilizes tight junctions, preserves cell polarity, and thereby, maintains epithelial barrier functions. Accumulating evidence since has shown that pharmacologic activation of AMPK by Metformin protects the epithelial barrier against multiple environmental and pathological stressful states and suppresses tumorigenesis. How AMPK protects the epithelium remained unknown until recently Aznar et al. identified GIV/Girdin as a novel effector of AMPK at the cell-cell junctions; phosphorylation of GIV at a single site by AMPK appears to be both necessary and sufficient for strengthening tight junctions and preserving cell polarity and epithelial barrier function in the face of energetic stress. Here we review the fundamentals of this specialized signaling pathway that buttresses cell-cell junctions against stress-induced collapse and discuss its pathophysiologic relevance in the context of a variety of diseases, including cancers, diabetes, aging, and the growing list of beneficial effects of the AMPK-activator, Metformin.

Epithelial cells usually display a polarized organization such that, localization of membrane proteins and positioning of organelles differ between the apical and basolateral sides of the cell [1]. Cell polarity is fundamental for both the architecture and function of epithelial tissues; its loss triggers organ dysfunction, neoplastic transformation and cancer progression, all via dysregulation of cell growth and division [2]. Epithelial polarization is established and maintained by a set of evolutionarily conserved signaling pathways, whose integration in space and time dictates overall epithelial morphogenesis [3]; together they collaborate to assemble, stabilize and turnover the cell-cell junctions, e.g. CDC42 and PAR proteins, such as the PAR3-PAR6-aPKC complex [4], and pathways that regulate membrane exocytosis and lipid modifications [4, 5].

## The stress-polarity pathway, a special force that resists junctional collapse during energetic stress

**Figure 1:**
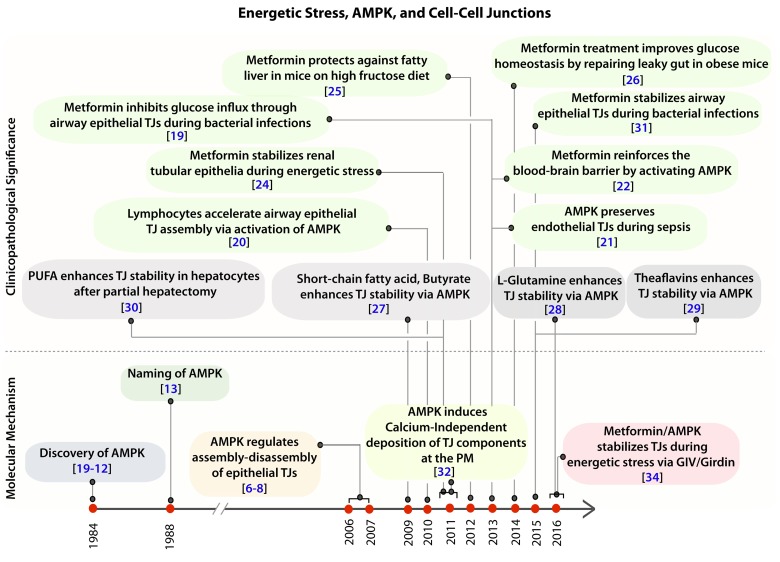
Clinical and pathologic significance of the protective role of AMPK in the epithelium during stress Schematic showing the time line of publications on the topic of AMPK and cell-cell junctions, as determined by a PubMed search in 2016, and their relationship to the recently published work by Aznar et al. [34]. *Top*: Clinical and pathological significance of pharmacologic activation of AMPK, either by the widely prescribed anti-diabetic drug, Metformin (green) or by other nutritional / dietary supplements (grey) in the regulation of tight junction stability and function. *Bottom*: Time line of publications unraveling the role of AMPK in the regulation of epithelial tight junctions and in the establishment of cell polarity.

Besides the pathways mentioned above, regulation of polarity requires an additional signaling component which is triggered exclusively under conditions of energetic stress. Three studies [6-8] published in 2006-07 simultaneously reported a surprising role of AMP-activated protein kinase (AMPK) in the maintenance of epithelial cell polarity and barrier functions (Figure 1). Discovered in 1984 [9-12], and named subsequently in 1988 [13], AMPK is unique in that it is a metabolic sensor protein which is activated exclusively during energetic stress. It is because of its ability to couple energy sensing to cell polarity, activation of AMPK was critical for protecting cell junctions against stress-induced collapse. Using polarized epithelial [Madin Darby Canine Kidney (MDCK)] cells it was demonstrated that AMPK is activated during calcium (Ca^2+^)-induced tight junction (TJ) assembly [6, 7]. The catalytic activity of AMPK is critical because either depletion of the AMPK catalytic α-subunit or expression of a kinase-dead mutant of AMPK inhibits TJ assembly as indicated by a loss of transepithelial electrical resistance (TEER); the latter is a measure of paracellular ion flow which depends on TJ stability. Pharmacological activation of AMPK with 5-aminoimidizole-4-carboxamide riboside (AICAR) partially protects TJs despite Ca^2+^ depletion [6, 7]. These findings closely followed another major revelation that the tumor suppressor LKB1 (Liver Kinase B1; also known as Serine/Threonine Kinase 11 - STK11) is a direct activator of AMPK [14-17], and that defects in cell polarity precede the development of tumors (pancreatic ductal adenocarcinoma) in genetically modified mice with tissue-specific deletion of LKB1 [18]. Together, these discoveries established the first links between energetic stress, cell polarity and oncogenesis. Since then, multiple studies (summarized in Figure 1) have reported the protective role of AMPK in maintaining cell-cell junctions across a variety of cell types in diverse tissues [airway and lungs [19, 20], heart [21], the blood-brain barrier [22, 23], kidney [24], intestine [25-29], liver [30]] while mounting a pathologic response to a variety of stressors, from bacterial invasion [31] to ischemia [24].

Although there is a wide consensus on the role of the LKB1-AMPK axis, and in particular AMPK's role in reinforcing TJs and preserving cell polarity during adverse environmental changes, how this kinase actually accomplishes this task, apparently in a Ca^2+^-independent manner [32], remained largely unknown until recently. One study suggested that muscle myosin regulatory light chain (MRLC) may be the effector of AMPK during energetic stress in the fly [8], but those findings have since come into question [33] because the phosphosites on MRLC do not conform to the optimal AMPK substrate motif found in all other established *in vivo* AMPK substrates. Thus, even though it had been a decade since the first studies revealed AMPK's ability to preserve the epithelial architecture and function in the setting of energetic stress, effectors of AMPK that orchestrate these functions had not been identified.

## The polarity scaffold, GIV, is a novel substrate and effector of AMPK within the stress polarity pathway

A recent study [34] demonstrated that GIV (G-alpha interacting vesicle associated protein, a.k.a. Girdin), a multimodular polarity scaffold protein is a novel substrate of AMPK, and defined the molecular mechanisms by which the AMPK-GIV signaling axis protects the epithelium by stabilizing TJs and preserving cell polarity when challenged with energetic stress. GIV, a guanine nucleotide exchange factor (GEF) for trimeric G proteins, had previously been shown to serve as a polarity scaffold protein that regulates epithelial cell polarity and morphogenesis [35-37]. GIV's role at cell-cell junctions has been attributed to its ability to assemble various functional complexes with its C-terminus, e.g., (i) binding the Par3/Par6/ aPKC polarity complex [36, 38]; (ii) binding and modulating the endocytic trafficking of E-cadherin [39]; (iii) linking cadherin-catenin complexes to the actin cytoskeleton [37]; and finally, (iv) binding and activating G protein, Gαi via its GEF motif and maintaining epithelial polarity through the Par polarity complex [36]. Each of these functional associations of GIV earned it the title of ‘polarity scaffold protein’ and have been implicated in the generation of cell polarity.

By demonstrating that GIV is a direct target and an effector of the energy sensing kinase AMPK, Aznar et al., [34] defined the stress polarity pathway at a greater resolution, nearly a decade after the discovery of the pathway. They showed that energetic stress triggers localized activation of AMPK at the tricellular TJs, which mark the most vulnerable cell-cell contacts in sheets of polarized cells. Activation of AMPK triggers phosphorylation at a single site within GIV, i.e., Ser(S)245. When phosphorylated by AMPK, pS245-GIV preferentially localizes to the bicellular and tricellular TJs. Such localization is seen exclusively during TJ turnover, i.e., localization is seen both during TJ assembly as cells come in contact to form a monolayer and during TJ-disassembly as monolayers collapse in response to energetic stress or Ca^2+^-depletion. Their findings also led to the conclusion that phosphorylation on GIV S245 is a key determinant of normal epithelial morphogenesis-- phosphorylation favors polarized normal cysts, whereas absence of phosphorylation favors branching tubules and multi-lumen structures that are associated with loss of cell polarity. Finally, they showed that pS245-GIV, which is generated only when the AMPK-GIV axis is intact, is both necessary and sufficient to fortify TJs, avoid junctional collapse and preserve cell polarity in the face of energetic stress, all in a Ca^2+^-independent manner. They further concluded that a significant part of the junction-stabilizing effects of AMPK agonists AICAR and Metformin during energetic stress [6, 7] are mediated by AMPK via its downstream effector, pS245-GIV. In demonstrating these, the authors revealed an elusive link between the stress-sensing components and the cell polarity pathways, and shed light onto how epithelial monolayers are protected despite being constantly bombarded by energetic stressors by fortifying cell-cell junctions against stress-induced collapse.

Mechanistically, they showed that pS245-GIV localizes to the TJ-associated microtubule tracks; 3D recons-truction of deconvolved confocal images revealed that pS245-GIV colocalized with and followed the bundles of polymerized microtubule tracks at the cell-cell borders, raising the possibility that the phosphoevent may impact GIV's ability to bind α- and/or β-tubulin heterodimers. Such localization appears to be facilitated by a direct interaction between the N-terminus of GIV [exclusively when phosphorylated at S245] and the short (∼100 aa) C-terminus of α-tubulin; the latter is known to project as helices from polymerized MT tracks [40, 41]. Once localized to the TJs, GIV may subsequently impact cell polarity and junctional integrity by assembling various aforementioned functional complexes with its C-terminus. Because AMPK regulates acetylation of the C-terminus of α-Tubulin during energetic stress [42] and because it is capable of stimulating microtubule polymerization at the cell periphery via phosphorylation of the microtubule plus-end protein, CLIP-170 [43], it is possible that either or both of these phenomena contribute to restricting the distribution of pS245-GIV exclusively at or near the junction-associated micro-tubule tracks.

It is also noteworthy that GIV's C-terminus (which binds Par complexes, G protein, and cadherin-catenin complexes), its N-terminally located AMPK substrate site, and α-tubulin-binding domain are highly conserved across all mammals and in birds; however, GIV lacks a consensus AMPK site in drosophila, and its C-terminus is poorly conserved in fish. These observations are consistent with others’ observation that the LKB1/AMPK stress polarity pathway is not evolutionarily conserved; it is not required for the maintenance of polarity during energetic stress in either flies [44, 45] or fish [46, 47] [no evidence exists in amphibians, reptiles, or birds], instead, the pathway is evolutionarily young, raising the possibility that it may have co-evolved with GIV to meet the metabolic demands of endotherms (birds and mammals).

## Pathophysiologic implications of the AMPK-GIV stress signaling pathway

**Barrier (dys)function**: Although the stress polarity pathway was originally demonstrated in polarized epithelial cells, studies using the AMPK activator, Metformin have demonstrated that AMPK fortifies cell-cell junctions in both epithelial [19, 24, 25, 31] and in endothelial cells such as those lining the lung alveoli [48], blood vessels [21] and the blood-brain barrier [22, 23, 49, 50] in the setting of stressors such as ischemia or sepsis (see Figure 1). Because GIV is ubiquitously expressed junctional scaffold, in both epithelial [36] and endothelial cells [39], it is possible that the stress-triggered mechanisms outlined by Aznar et al., [34] enable the barrier-protective role of AMPK at TJs observed in a diverse organs and tissues, both epithelial and endothelial linings, when challenged with chemical, bacterial and metabolic stressors (Figure 1).

Among the different body cavity linings (barriers), the mucosal barrier where the stress polarity pathway may be of greatest relevance is the intestinal mucosa. This barrier represents a huge mucosal surface, which separates billions of bacteria from the largest immune system of the body. On the one hand, the TJs of an intact intestinal barrier protect us against potential barrier disruptors, e.g., hypoperfusion of the gut, microorganisms and toxins, over-dosed nutrients [high fat], drugs, and other elements of lifestyle. On the other hand, this barrier must be open to absorb essential fluids and nutrients. Over the years, the beneficial [protective] effect of multiple nutritional components, dietary supplements, and pharmacologic agents, including the widely-prescribed AMPK-activator, Metformin on intestinal permeability in health and disease has been investigated; all studies converge on AMPK activation as a common pre-requisite for rendering such protection (see Figure 1). These studies raise the possibility that the AMPK-GIV stress polarity pathway defined by Aznar et al., may affect a variety of diseases that are associated with increased intestinal permeability (reviewed in [51]) such as critical illness, inflammatory bowel diseases [52, 53], celiac disease, food allergy, irritable bowel syndrome [54, 55], Alzheimer's [56], Parkinson's [57], multiple sclerosis [58-60], autism [61, 62], chronic heart failure [63-65], aging (expanded below) and obesity and metabolic diseases (expanded below). All these diseases are characterized by systemic inflammation due to chronic endotoxemia that might be triggered by the translocation of endotoxins from the gut lumen into the host circulation.

**Figure 2:**
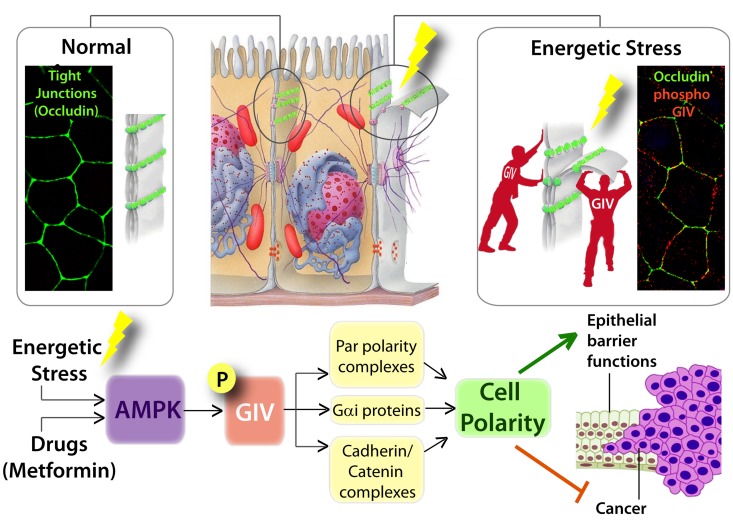
Graphical abstract summarizing how AMP-activated protein kinase fortifies epithelial tight junctions during energetic stress via its effector GIV/Girdin Schematic showing the pertinent findings reported in by Aznar et al. [34]. *Top (from left to right)*: In normal physiologic states, sheets of polarized epithelial cells maintain barrier integrity by assembling tight junctions TJs; stained here with the TJ-marker and integral membrane protein, Occludin in green. Exposure to energetic stress triggers the activation of AMPK, a sensor of cellular energy stores, which in turn phosphorylates GIV at Ser245. Phospho-GIV [stained red] localizes to the TJs [marked with occluding] and serves to stabilize TJs and resist stress-induced collapse. *Bottom*: Schematic summarizing how the AMPK-GIV signaling axis preserves TJ integrity via multiple interacting partners of the polarity scaffold, GIV, and how this stress-polarity pathway enhances barrier functions and inhibits neoplastic transformation.

**Cancers**: Previous work has shown that polarity defects precede the onset of tumorigenesis when the LKB1-AMPK pathway is inhibited (demonstrated in mice lacking the tumor suppressor and AMPK activating kinase, LKB1; [18]). These findings had fueled speculation that polarity defects may be one of the major mechanisms for tumor initiation when the energy sensing pathway is dysregulated [66]. Aznar et al., [34] showed that the AMPK-GIV stress-polarity pathway inhibits oncogenic transformation and growth, and that disruption of this pathway (accomplished via mutations identified during genomic sequencing of colorectal cancers) helps tumor cells escape such inhibition and gain proliferative advantage during 3D growth (Figure 2). Because LKB1 is a master kinase that can activate all 13 members of the AMPK kinase family [67], and given the overlapping substrate specificity of AMPK and its related kinases (reviewed in [33]), it seems likely that AMPK-related family members, such as MARK/Par1, may phosphorylate S245 on GIV under specific conditions and in certain cancers. For example, in the case of gastric cancers, where elevated GIV expression carries poor prognosis [68], junctional/ polarity defects are often observed. In this cancer, the carcinopathogen *H. pylori* drives cell transformation by delivering its virulence factor CagA (cytotoxin-associated gene A) into gastric epithelial cells through a bacterial type IV secretion system [69]. Upon entering the epithelial cells, CagA specifically binds and inhibits MARK/Par1 polarity kinase, triggering junctional and polarity defects [70]. It is tempting to speculate that MARK/Par1 may phosphorylate GIV at S245, and that inhibition of MARK/Par1 by CagA could deregulate the MARK-GIV signaling axis, thereby heralding neo-plastic transformation. Future studies are planned to investigate if such is the case.

**Obesity, metabolic syndrome and type II diabetes**: Accumulating evidence shows that gut barrier dysfunction can influence whole-body metabolism [71, 72] by affecting the energy balance [71], gut permeability [73, 74], metabolic endotoxemia [75] and inflammation [72, 73, 75, 76] that are associated with obesity and the spectrum of disorders associated with metabolic syndrome [25, 77, 78]. Numerous studies using the AMPK-activator, Metformin, squarely implicate the AMPK-dependent stress polarity pathway as a major therapeutic target in these metabolic disorders [79-81]. Metformin administration enhances gut barrier integrity, attenuates endotoxemia and enhances insulin signaling in high-fat fed mice, which accounts for the beneficial effects of metformin on glucose metabolism, enhanced metabolic insulin response, and reduced oxidative stress in liver and muscle of the mice [79]. Clinical trials using a delayed release formulation of Metformin (Metformin DR, which is designed to target the lower bowel and limit absorption into the blood) have shown that metformin works largely in the colon; despite the reduced levels of absorption of Metformin DR, this formulation was effective in lowering blood glucose [81]. Metformin treatment directly impacts the colonic mucosa and the gut microbiome [26]; the number of goblet cells and mucin production increases, senescence is reduced, and *Akkermansia muciniphila*, which is a mucin-degrading bacterium that resides in the mucus layer becomes abundant. Others have demonstrated that the presence of this bacterium directly correlates with gut barrier integrity [80, 82] and inversely correlates with body weight and visceral adiposity in rodents and humans [80]. These studies have challenged the conventional thinking and the importance of the gut barrier as the primary defect in metabolic diseases has gained traction [83-85]. These studies also highlight the effectiveness of activation of AMPK as a therapeutic strategy to reinforce the gut barrier and correct metabolic disorders.

**Aging**: Aging is characterized by the functional decline of individual organ systems of an organism, and progressively increases the probability of death. Among the various organ systems that decline during aging, dysfunction of the intestinal barrier has been correlated with increasing age in a variety of species. For example, dysfunction of the intestinal barrier predicts impending death in individual flies regardless of the chronological age [86]. Much like humans, these flies show an age-related increase in immunity-related gene expression (e.g., IL6) accompanies such dysregulation of barrier [86]. Evidence also shows that intestinal barrier dysfunction during aging is conserved in worms (*C. elegans*) and fish (*D. rerio*) [87, 88], and in mammals (rats [89] and baboons; [90]), thus raising the possibility that it may also be the case in humans. However, studies in humans have shown that intestinal permeability is not increased simply due to aging, but increases in the setting of coexisting stressors such as low-grade inflammation and/or type II diabetes [91].

As for the mechanism of increased permeability, colonic biopsies from aging baboons showed that increased permeability is associated with age-associated remodeling of epithelial TJs (decreased zonula occluden-1, occludin, and junctional adhesion molecule-A tight junction protein expression and increased claudin-2 expression; the latter promotes the formation of pores that allow the paracellular movement of cations and small molecules and increases permeability) [90]. In fact, several important physio-logical processes that are dependent on TJ integrity and cell polarity are altered during aging, involving both epithelial and endothelial cells (reviewed in [92]). It is possible that the observed anti-ageing properties of Metformin (via multiple widely pleiotropic effects reviewed in [93]), as in the case of obesity and diabetes, may begin by preserving the gut barrier function, thereby reducing age-related inflammation and metabolic derangements. If so, Metformin is expected to act via the AMPK-GIV stress polarity pathway to resist aging related increase in gut permeability. Ongoing clinical trials approved by the FDA (such as Targeting Ageing with Metformin; TAME) are likely to provide the best opportunity to investigate these possibilities.

**Mechanism of action of the wonder drug, metformin**: For almost a century, ever since the biosynthesis of the xenobiotic metformin by Emil Werner and James Bell in 1922, scientists have been revisiting the mechanism of action of this first-line treatment for type II diabetes. Metformin (Glucophage) is now the most widely prescribed type II diabetes drug in the world; it reduces blood glucose by activating the LKB1-AMPK pathway [94] and inhibiting hepatic gluconeogenesis (reviewed in [33]). Besides its ability to lower blood glucose, Metformin also exerts two other effects in an AMPK-dependent manner: (i) it stabilizes cell-cell junctions and protects barrier functions of both epithelial and endothelial monolayers in the setting of a variety of pathologic stressors; and (ii) it suppresses the growth of a variety of tumor cells and embryonic stem cells in culture and tumor xenografts in mice [reviewed in [33]]. By demonstrating that phosphorylation of GIV by AMPK is required for Metformin to exert both these effects efficiently, Aznar et al., [34] implicated the AMPK-GIV signaling axis as an important mechanism of action of Metformin. It is noteworthy that although multiple retrospective clinical trials have generally concluded that prolonged use of Metformin reduces the incidence of cancer, others have reported conflicting results, and several prospective clinical trials are underway to identify which target populations may specifically benefit from this drug (reviewed in [95, 96]). Given the widespread long-term use of metformin as a prescription drug and its potential utility both in chemoprevention as well as chemotherapy, further studies are warranted to investigate if the GIV-expression status in tumors (e.g., its expression as a spliced isoform lacking the C-terminus [97] or mutants that prevent phosphorylation by AMPK [34], or its overexpression as full length [98]) may help identify which patients may benefit from the tumor suppressive actions of the Metformin.

In conclusion, just when investigations on the phenomenon of stress polarity pathway had hit a cold trail, findings reported by Aznar et al. have reopened the topic by netting a strong set of clues (GIV) and have raised many more important questions. Also remains unknown how the interplay between the newly discovered AMPK→GIV signaling axis with multiple other inputs and outputs within the AMPK energy-sensing pathway (reviewed in [99, 100]), with the plethora of interactions within the expanding AMPK interactome [101], with newly emerging substrates within new pathways, e.g., neucleosome modeling [102], the glycolytic pathway [103], mitochondrial dynamics [104], junctional scaffolds, like cingulin [105], regulators of microtubule dynamics, like CLIP 170 [43]. Future studies are warranted to seek answers to these questions so that the pathophysiologic implications of this pathway and its potential as therapeutic target in a plethora of chronic diseases can be fully realized.
